# Leachate Pollution Index (LPI) in Sanitary Landfills in the High Andean Zones of Peru

**DOI:** 10.3390/molecules30163325

**Published:** 2025-08-08

**Authors:** Liliana Rodriguez-Cardenas, Yudith Choque-Quispe, Aydeé M. Solano-Reynoso, Diego E. Peralta-Guevara, Yakov F. Carhuarupay-Molleda, Henrry W. Agreda-Cerna, Odilon Correa-Cuba, Ybar G. Palomino-Malpartida, Yovana Flores-Ccorisapra, Delma D. Reynoso-Canicani, Jorge W. Elias-Silupu, Luis H. Tolentino-Geldres, David Choque-Quispe

**Affiliations:** 1 Water and Food Treatment Materials Research Laboratory, Universidad Nacional José María Arguedas, Andahuaylas 03701, Peru; amsolano@unajma.edu.pe; 2 Environmental Engineering Department, Universidad Nacional José María Arguedas, Andahuaylas 03701, Peru; ychoque@unajma.edu.pe; 3 Department of Basic Sciences, Universidad Nacional José María Arguedas, Andahuaylas 03701, Peru; ycarhuarupay@unajma.edu.pe (Y.F.C.-M.); odiloncorrea@unajma.edu.pe (O.C.-C.); 4 Agroindustrial Engineering Department, Universidad Nacional José María Arguedas, Andahuaylas 03701, Peru; deperalta@unajma.edu.pe; 5 Research Group for the Development of Advanced Materials for Water and Food Treatment, Universidad Nacional José María Arguedas, Andahuaylas 03701, Peru; 6 Department of Business Administration, Universidad Nacional José María Arguedas, Andahuaylas 03701, Peru; hagreda@unajma.edu.pe; 7 Chemical Engineering Department, Universidad Nacional de San Cristobal de Huamanga, Ayacucho 05000, Peru; ybar.palomino@unsch.edu.pe; 8 Systems Engineering Department, Universidad Nacional José María Arguedas, Andahuaylas 03701, Peru; yflores@unajma.edu.pe; 9 Academic Department of Accounting and Finance, Universidad Nacional José María Arguedas, Andahuaylas 03701, Peru; dreynoso@unajma.edu.pe; 10 Department of Science and Engineering, Universidad Nacional Ciro Alegria, Huamachuco 13301, Peru; jelias@unca.edu.pe (J.W.E.-S.);

**Keywords:** leachate, leachate pollution index (LPI), landfill, biodegradability

## Abstract

Cultural customs often condition solid waste management, especially in developing countries. The decomposition of solid waste depends on climatic conditions and is related to geomorphology and anthropogenic practices. Leachate generated in landfills can migrate superficially and underground, contaminating soils and aquifers. Knowing the level of contaminant load in leachate is important for proper solid waste management. However, in the Andean regions of Peru, there is scarce data on the polluting potential of leachates. This research aimed to determine the Leachate Pollution Index (LPI) according to the sub-indexes of organic, inorganic, and heavy metals from landfills in the high Andean regions of Peru. Physical, chemical, and microbiological parameters were evaluated in fresh and retained leachate samples, in both dry and rainy seasons, from two landfills located at around 3000 m of altitude. The results showed high contamination levels, particularly in BOD_5_, COD, NH_3_-N, and total coliforms, with high organic and inorganic sub-indexes that affect the LPI, indicating high levels of contamination and posing a potential risk to surrounding ecosystems. It was also found that the high Andean landfills studied have a good-to-high biodegradability. This research contributes essential baseline information for environmental monitoring and supports the need for improved leachate management in high-altitude landfills in Peru and similar Andean contexts.

## 1. Introduction

The prevention and proper management of solid waste are essential to ensuring a sustainable future for generations to come [1]. Sanitary landfills are the most used option for solid waste management and disposal globally [2], especially in the developing countries of Latin America [3]. However, these sources contribute to the emission of gas and leachate [4], which are highly polluting and harmful to the surrounding ecosystem.

Leachate is produced from the moisture content present in the deposited waste and the rainwater that percolates through it [5]. Many factors influence its composition, such as the nature and moisture content of waste dumped, the particle size, the degree of compaction, the age of the dumpsites/landfills, the hydrology and climatic conditions of the site, and other factors [6,7]. It has been demonstrated that leachates are harmful to the environment [1,3,8,9,10,11,12,13], as they are considered to be one of the main threats to water sources [2]; in addition, modern sanitary landfills have been reported to demonstrate leachate leaks that contaminate the surrounding groundwater surface [14], due to the high concentration of organic and inorganic compounds and heavy metals present. In addition to these traditional pollutants, the presence of emerging contaminants in leachate—such as pharmaceuticals, microplastics, and hormonal compounds—has also been reported. Although not currently regulated, they pose significant environmental risks. Including them in future studies is essential for a more comprehensive assessment of leachate impact.

In Perú, there are currently 58 sanitary landfills [15] and more than 1585 waste dumps [16] that operate without any environmental protection, and there are no specific regulations or protocols for monitoring leachate [17]. In the provinces of Andahuaylas and Chincheros, waste generation is a problem [18], as its accumulation in landfills contributes to the production of leachate. The sanitary landfill in the Ancco-Huayllo district, Apurimac, Peru, lacks a leachate treatment system. In contrast, the landfill in the city of Andahuaylas has only a pumping system to recirculate leachate to the landfill cells.

The cities of Andahuaylas and Ancco-Huayllo are situated in the high Andean zones of Peru, above 3000 m in altitude. This location affects the leachate’s generation and quality due to this ecosystem’s characteristics. Abundant rain or frost increases the availability of water, while low temperatures may also slow down the decomposition of solid waste. For the planning, design, and management of sanitary landfills, it is important to consider the climatic and topographic characteristics of the site for leachate control [2,19]. Being high-altitude sites, they are part of the hydrographic basin, providing water for human supply and irrigation, as they are close to the headwaters of the basin. The geomorphology of the site may prove to be an ally in the infiltration of leachate and the contamination of water sources.

To assess the impact of leachates on the environment, their physicochemical and microbiological composition is analyzed. Several methodologies allow for the determination and evaluation of the degree of contamination, including analytical techniques and numerical methods [20]. The Leachate Pollution Index (LPI) is a quantitative tool designed to provide measurable data on leachate pollution from landfills. In this study, the LPI method was applied to assess and compare the pollution potential of two active sanitary landfills located in the high Andean region of Peru—Andahuaylas and Ancco-Huayllo. Sampling was conducted during the rainy and dry seasons, and leachate samples were analyzed to determine physicochemical parameters. The resulting data were used to calculate the LPI and evaluate seasonal and structural influences. This analysis serves as an environmental indicator to support decision-making. Therefore, understanding and quantifying the potential for leachate pollution is essential to selecting appropriate and effective processes for leachate management, handling, and treatment.

## 2. Results and Discussion

### 2.1. Seasonal and Temporal Variations

The average monthly temperature was 20 °C, with minimal variation throughout the year. The monthly rainfall in the province of Andahuaylas for the period from 1970 to 2019 ranges between 7.28 and 134.30 mm/month, with an annual average value of 686.73 mm/year. While for the province of Chincheros, this value ranges from 3.17 to 116.44 mm/month, with an average of 626.04 mm/year.

**Figure 1:**
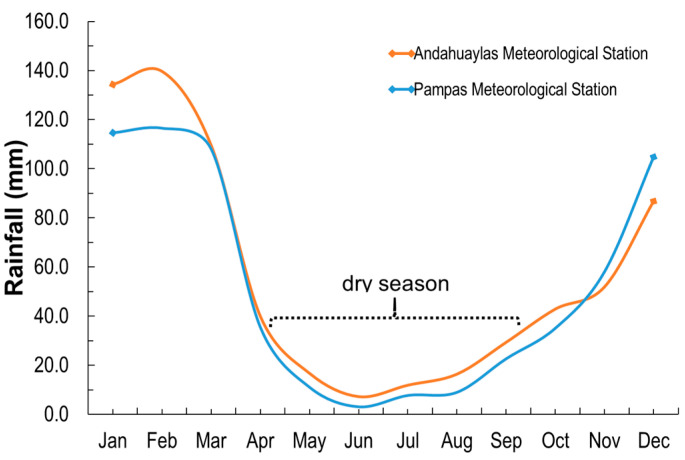
Average monthly rainfall for the period from 1970 to 2019.

The rainy season occurs between November and April, with average maximums in January and February that are close to 136 mm/month (Figure 1). The dry season is between May and October.

### 2.2. Estimation of Leachate Production

Table 1 shows the leachate discharge volumes; ASL registered an annual volume of 2565.95 m^3^ for an annual precipitation of 686.73 mm. Similarly, AHSL reported a lower volume of leachate (711.05 m^3^) for an annual precipitation of 626.04 mm, although with a smaller landfill area (6900 m^2^), indicating that the amount of leachate produced depends on precipitation, landfill surface, and the moisture content of the waste [21]. On the other hand, young landfills with 5 years of operation present leachates with a high organic load and dissolved solids that are harmful to the surrounding areas and water sources [22,23].

### 2.3. Evaluation of Physical, Chemical, and Biological Parameters of Leachate

The pH values of leachate from landfills were higher than 7.27, increasing in rainy seasons up to values of 8.45 (Table 2 and Table 3). These results are characteristic of landfills with an operating time of more than 10 years [24,25]. However, the AHSL and ASL have an operational life of approximately 5 years and should report a pH between 6.5 and 7.5 [24,25,26,27,28].

The elevated pH values are due to the recirculation of the leachate and the high precipitation causing a dilution of the pH, reducing the acidity. Likewise, the decomposition of solid waste produces ammonia, which forms ammonium and hydroxyl ions, raising the pH levels [29]. Conversely, a pH value exceeding 7 indicates a high alkalinity in the leachate, which may potentially impact the acidity of nearby water bodies, including surface and groundwater, which could have negative effects on aquatic life and ecosystems [29].

The TDS reported values between 7784.00 and 8621.00 mg/L for ASL (Table 2), and 6242.70 to 7614.6 mg/L for AHSL (Table 3), showing an increase during the rainy season. High values indicate a high degree of mineralization [1], as well as the presence of organic and inorganic substances dissolved in the leachate [1,30], which significantly altered the physicochemical characteristics of the receiving water, decreasing the clarity of the water with the consequent restriction of photosynthesis and growth of microorganisms [31,32,33].

The COD is a measure of the oxygen required for the complete oxidation of organic compounds present in waste materials [1]. The ASL reported values between 5016.80 and 9446.70 mg O_2_/L (Table 2), while the AHSL reported 9125.0 to 10,653.3 mg O_2_ /L, with higher COD concentration in rainy seasons. This is likely due to the water allowing the dissolution of soluble organic and inorganic matter [13,34,35]. High ASL and AHSL values would indicate the onset of an acidogenic phase in the leachate, where about 95% of the COD content is made up of volatile fatty acids (VFA) [36,37]. The landfills that report COD between 3000 and 15,000 mg O_2_/L are known as intermediate landfills with at least 10 years of operation [24,27,38]. Higher levels of COD significantly alter the physical properties of groundwater and surface water [39].

BOD_5_ measures the amount of oxygen required by the microorganism to decompose the organic pollutant [39]. The ASL presented BOD_5_ values between 2470.00 and 5960.00 mg O_2_ /L, while the AHSL was between 4720 and 7840 mg O_2_ /L, reporting high values in rainy seasons. These results are characteristic of the acidogenic phase, which varies between 4000 and 40,000 mg O_2_ /L. This would be due to the high organic content and great microbiological activity [40,41,42].

The ratio of BOD_5_/COD in ASL ranges between 0.4 and 0.6 (Table 2), while in ASRH it varies from 0.5 to 0.7 (Table 3). These results are characteristic of moderately stable fillings (between 0.1 and 0.5) and young fillings (greater than 0.5) [28], in addition, values above 0.4 would indicate that the leachate is in an acid phase, likewise, the ratio of BOD_5_/COD decreases as sanitary landfills age [1], due to the rapidly decreasing BOD_5_ and disintegration of biodegradable waste [30]. The values ranging from 0.6 to 0.75 [42] and from 0.4 to 0.5 of BOD_5_/COD [43] indicate the presence of biodegradable organic matter and some recalcitrant substances.

Total coliforms (TC) play an important role in the decomposition of organic and inorganic wastes. High TC values of 41,600 CFU/ 100 mL were reported in AHSL, and values below 1780 CFU/ 100 mL in ASL. The low TC values are due to the high salinity content in the leachate limiting bacterial growth [44]. During rainy seasons, the TC value increases considerably due to the percolation of water through the deposited waste [13]. Temperature also plays an important role since the decomposition of organic matter within the landfill can generate heat, giving rise to high temperatures in the leachate and increasing microbial activity.

NH_3_N is the most significant contaminant in long-term leachate, because it has an increasing trend over time, increasing due to the decomposition of nitrogenous organic matter and evaporation [23]. High levels of NH_3_N are often toxic to plants and animals, can contaminate groundwater, and contribute to eutrophication, which is a phenomenon that causes excessive growth of algae in groundwater [45]. On the other hand, NH_3_N control guarantees effective treatment of the leachate. It was observed that the ASL leachate ranges from 213.3 to 1243.3 mg/L (Table 2), and in AHSL between 983.3 and 1810.0 mg/L (Table 3). On the other hand, the pH conditions its presence, since at high values, it causes the nitrogen to be in its ionic form giving an increase in NH_3_N [46].

The highest Cl^−^ concentration was reported in ASL at 3220 mg/L during dry seasons (Table 2), and the lowest value was reported in AHSL at 660 mg/L during rainy seasons (Table 3). Some waste, such as aerosols, deodorants, shampoos, moisturizing creams, and disinfectants, release Cl^−^ when decomposing. In addition, Cl^−^ levels of up to 3890 mg/L have been observed [13,35,47]. High levels of Cl^−^ are toxic to aquatic microorganisms; on the other hand, it can increase the mobility and bioavailability of toxic heavy metals in the soil, increasing the risk of contamination and bioaccumulation in the food chain [48].

On the other hand, cyanide (CN^−^) is a compound found in various products such as batteries, plastics, pesticides, and cleaning products [49]. The highest value of CN^−^ was reported in ASL with 0.063 mg/L in rainy seasons (Table 2), because the water increases the temperature and humidity of the landfill, causing the release of CN^−^ from the waste [50]. High levels of CN^−^ are toxic to water sources [51].

The most common metals in the leachate are As, Cd, Cr, Co, Cu, Pb, Hg, Ni, and Zn [52]. The analyses show that most metals in the leachate have a concentration below 0.05 mg/L, except for As and Fe, which are above 1.25 mg/L in both landfills during rainy and dry seasons (Table 2). Several studies indicate that metal concentrations are generally low in many landfills and dumpsites, apart from Fe [28,36,53]. However, As is present in the leachate in both organic and inorganic forms [54]. The landfills under study are influenced by nearby copper and iron mining areas, which could also introduce lead and arsenic.

### 2.4. Leachate Pollution Index (LPI)

The LPI_or_ composed of COD, BOD, and CT, determines the biodegradability of the leachate [55]. ASL reported values between 68.39 and 57.65 (Table 4), while AHSL was between 77.53 and 64.99 (Table 5), being higher during the rainy season for both landfills. In both cases, they exceed the standard value for the LPI_or_ of 7.03. This would be due to the high concentrations of organic matter in the leachate, characteristic of an acidogenic phase [42]. This behavior is characteristic of developing countries, which have high LPIor values [39,56,57], because 60% of the waste it generate is organic waste [3,55].

The LPI_in_ values in the rainy season are higher than 14.48, while in dry seasons, values are reported above 26.52 (Table 4 and Table 5). This is due to the low water levels in the landfill, causing an increase in the leachate concentration. In addition, the bacteria that decompose organic matter are less active in dry seasons because they need water to survive, generating an increase in NH_3_N. The LPI_in_ values have been reported in active and closed landfills, where the main inorganic pollutants were total nitrogen and NH_3_N, which favor an increase of 38% to 41% of the LPI_in_ [10,59]. Likewise, the concentrations of Cl^−^, NH_3_N, and TDS are high in dry seasons [60]. The ASL and AHSL results exceed the standard value of LPI_in_ of 6.57 [59].

Low concentrations of heavy metals help the growth of microorganisms and the biological treatment of leachates because metals are toxic to microorganisms, hindering their growth [42]. The ASL and AHSL reported values for the LPI_hm_ below 11.45, although these are higher than the standard value of 7.89 [59]. This would be due to the high organic load and low concentration of metals in the leachate [42]; in the dry season, it is higher [60].

#### LPI Overall (LPI_ov_)

The decomposition of organic waste within the landfill increases the concentrations of COD, BOD, and microbial activity. Therefore, high concentrations of organic components have a greater effect on increasing the LPI_ov_ [1]. The ASL and AHSL reported values above 24.80. It was observed that AHSL reported the highest value (30.88) in the rainy season (Table 5), while the highest value was reported in the ASL during the dry season (29.75) (Table 4), due to high concentrations of BOD, COD, and dissolved metals such as As, Cr, and Fe. Likewise, the LPI_ov_ is significantly influenced by the physicochemical parameters of the leachate [39]; although, in the rainy season, some contaminants such as pH, TDS, and Cl^−^ are diluted by the rain, causing low concentrations [61]. On the other hand, high rainfall in humid climates has a significant effect on COD, total carbon, TC, NH_3_N, and heavy metals [21,62].

The fillers under study exceed the standard LPI_ov_ of 7.37 [55,59], which is classified as landfills that generate highly contaminated leachates. This same behavior has been observed in developing countries, where LPI_ov_ values significantly exceed the standard threshold (Table 6) [3,10,39,53,59,60,63,64,65,66,67], which is why measures must be taken to prevent environmental contamination [1], as well as to propose management and segregation policies at source, in addition to information campaigns on solid waste management and the impact of leachates on water bodies.

On the other hand, the structural differences between the ASL and AHSL contribute to the variations observed in LPI values. The ASL is equipped with a bottom liner and a leachate recirculation system, which help retain and manage leachate within the landfill. In contrast, the AHSL lacks both systems, allowing for the greater infiltration of leachate into surrounding soils and increased percolation during the rainy season. This structural difference affects the dilution of contaminants such as pH, TDS, and Cl^−^, as well as the mobilization of heavy metals. During the dry season, the absence of recirculation may lead to the accumulation of more concentrated leachate, which explains the higher LPI_in_ and LPI_hm_ values observed. These findings highlight the importance of proper landfill design in controlling leachate pollution.

### 2.5. Correlation and Principal Component Analysis

**Figure 2:**
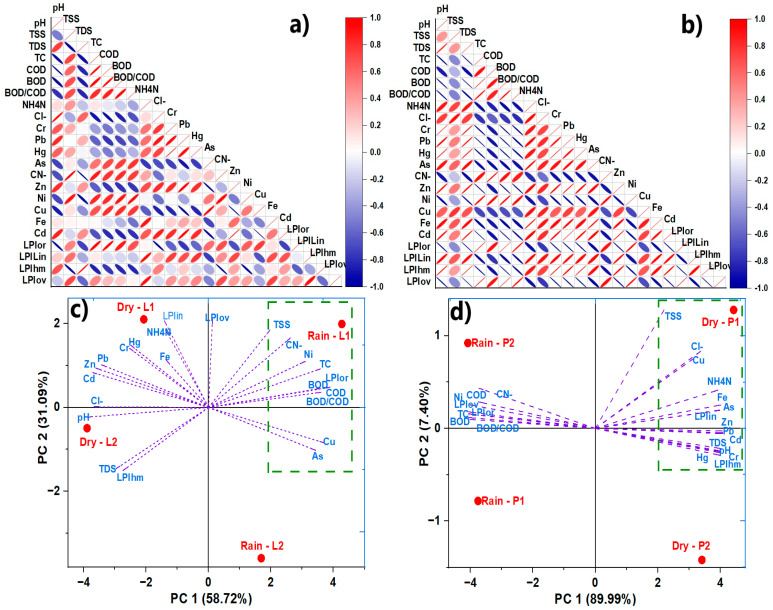
Pearson’s correlation and principal component analysis. Bivariate Pearson’s correlation analysis (**a**,**b**) and principal component analysis (**c**,**d**) of leachate parameters, sub-indices, and the LPI of ASL and AHSL.

According to Pearson’s correlational analysis of the ASL leachate characterization, LPI_or_ showed a significant and positive correlation with the parameters TC, COD, and BOD_5_, which coincides with the presence of organic matter in the leachate and had significant negative correlations with pH and Cl^−^. However, LPI_in_ in the leachate was significantly and positively correlated with NH_3_-N, Cr, Pb, and Hg, as well as negatively with As. In addition, LPI_hm_ was significantly and positively correlated with TDS and negatively correlated with TSS, CN^−^, and Ni. Finally, it is observed that LPI_ov_ was positively related to LPI_or_ and LPI_in_ and was negatively correlated with LPI_hm_ (Figure 2a).

In AHSL, the LPI_or_ was positively correlated with the parameters TC, BOD_5_, BOD_5_/COD, and Ni, while it was negatively correlated with pH, TDS, Cr, Pb, Hg, Zn, Fe, and Cd. On the other hand, the LPI_in_ was positively correlated with pH, TDS, NH_3_-N, Cl^−^, Cr, Pb, Hg, As, Zn, and Cd, and negatively correlated with TC, BOD_5_, BOD_5_/COD, and Ni. Likewise, the LPI_hm_ was significantly and positively related to several metals such as Cr, Pb, Hg, As, Zn, Cd, Cu, and Fe (Figure 2b). On the other hand, the LPI_ov_ was positively related to LPI_or_ and negatively related to LPI_in_ and LPI_hm_.

The significant correlations observed may indicate trends or groupings of the dominant leachate parameters in the sanitary landfills. One way to verify this behavior is through a principal component analysis (PCA) approach, which provides a visual and preliminary insight into the variability within the sampling areas.

In the principal component analysis (PCA) for ASL, the accumulated variance of PC1 and PC2 components was 89.81% of the total data variance. Most of the chemical parameters are positively related; pollutants such as TSS, CN^−^, Ni, TC, BOD_5_, COD, and LPIor are associated with the organic load of the leachate, indicating that it has a greater influence in the rainy season in L1. In the ASL, rainfall seems to have a significant effect on the composition of the leachate, which is probably due to dilution and changes in chemical and biological processes. On the other hand, heavy metals (Zn, Pb, Cr, Hg, Fe, and Cd) and pH are more influential or have a greater presence in dry climatic conditions, indicating that in dry seasons, evaporation and metal concentration are evident (Figure 2c).

For AHSL, the combined variance of the PC1 and PC2 components was 97.39%, with PC1 explaining 89.99% and PC2 explaining 7.40% of the data’s variation. The most significant and influential pollutants during the dry season are TSS, Cl^−^, Cu, NH_3_N, Fe, and As, indicating that evaporation and decreased water volume in the leachate lead to higher concentrations of organic pollutants and metals in the AHSL (Figure 2d).

The PCA reveals a clear distinction between the rainy and dry seasons regarding the dominant types of pollutants. The rainy season is characterized by a higher organic load, while the dry season shows a greater concentration of inorganic pollutants and heavy metals (Figure 2c,d).

### 2.6. Treatment Proposals

The implementation of a treatment for leachate in landfills must take into account several factors, including the volume of leachate, its chemical composition, the design of the landfill, and the climatic conditions of the area. Some treatment strategies for leachate include biological treatments, which are commonly used for young leachates with a high BOD_5_/COD ratio, as they help degrade organic matter; however, their effectiveness decreases with the presence of refractory compounds. Physicochemical methods like coagulation, chemical oxidation, and adsorption are useful when biological treatments are not sufficient. Co-treatment with domestic wastewater is low-cost and widely used, but the presence of toxic substances in leachate may reduce the efficiency of the treatment plant. Advanced technologies, such as membrane filtration (e.g., nanofiltration or reverse osmosis), allow for the removal of a wide range of pollutants, including emerging contaminants, but they require higher investment and maintenance. Another option is leachate recirculation within the landfill, which is inexpensive and improves waste stabilization, although it may cause odor or leachate accumulation if not properly managed. The choice of treatment should be based on site conditions, pollution level, and long-term environmental impact.

Another important aspect is the commitment of government authorities to implement monitoring systems both inside and outside the landfill, as well as the continuous assessment of nearby water bodies, which may be at risk due to leachate infiltration. Although regulations exist, many lack a solid scientific basis and are often not effectively enforced.

## 3. Materials and Methods

### 3.1. Study Area

**Figure 3:**
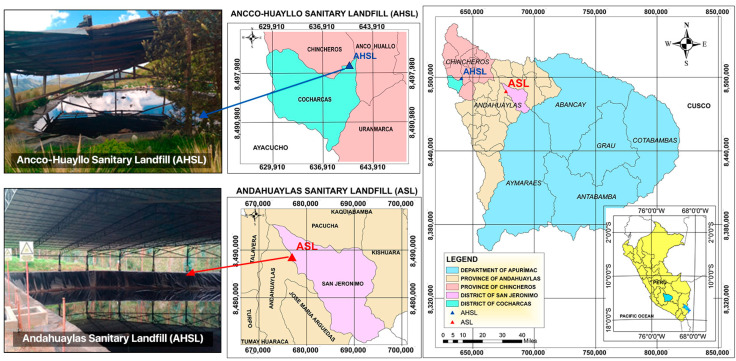
Study area location.

The Andahuaylas Sanitary Landfill (ASL) site is situated in Cerro San José in the district of San Jerónimo, Andahuaylas, Peru, with coordinates 13°39′50.67″ S and 73°21′50.50″ W (Figure 3); it commenced operations in 2019 and receives about 24.31 tons of municipal solid waste per day [18] from the districts of Andahuaylas, San Jerónimo, Talavera, and Pacucha.

The Ancco-Huayllo Sanitary Landfill (AHSL) is situated in the district of Cocharcas, in the province of Chincheros, Apurímac, Perú, with coordinates 13°34′23.50″ S and 73°42′0.83″ W (Figure 3). This landfill is approximately 5 years old and receives 6.46 tons of solid waste per day from the district of Ancco-Huayllo [68].

Table 7 shows the current information on the Andahuaylas and Ancco-Huayllo Sanitary Landfills [69]. The ASL and AHSL are contemplated for the disposal of municipal solid waste with an operational stage of 10 years. Table 8 shows the total amount of solid waste deposited in the sanitary landfills in 2022, with a greater contribution from the district of Andahuaylas.

### 3.2. Sample Collection and Analysis

#### 3.2.1. Sampling and Preservation of Leachate

Samples were collected from two leaching ponds belonging to the ASL (L1 and L2) and AHSL (P1 and P2); for each point, a composite sample was obtained by mixing aliquots taken from different areas and depths of the respective pond in order to better represent the spatial variability of the leachate. Sampling was conducted during the rainy season (April 2023) and the dry season (June 2023). Sampling was carried out in April 2023 (rainy season) and June 2023 (dry season). April was selected to capture the influence of heavy rainfall and runoff from previous months (particularly February), while June was chosen to reflect dry conditions with low precipitation. A volume of 2 L of leachate was considered in each study period. Samples were collected in previously sterilized one-liter (1 L) bottles, and sterile Whirl-Pak bags were utilized for microbiological analysis, according to the protocol established by AWWA [70]. The samples were stored at 4 °C and transported to the laboratory within 24 h for analysis.

#### 3.2.2. Analysis of Samples

The collected leachate samples were analyzed for physical, chemical, and microbiological parameters, as specified in Table 9. Likewise, the leachate samples were filtered at 0.45 μm to determine the iron (Fe) (λ = 239.14 nm), copper (Cu) (λ = 205.49 nm), cadmium (Cd) (λ = 214.43 nm), zinc (Zn) (λ = 202.54), lead (Pb) (λ = 220.35), total chromium (Cr) (λ = 205.55), mercury (Hg) (λ = 184.95 nm), and arsenic (As) (λ = 189.04 nm) content using Inductively Coupled Plasma–Optical Emission Spectrometry (ICPE-9820, SHIMADZU, Tokyo, Japan). Leachate samples were analyzed in triplicate, in axial mode, at 60 rpm between samples, with a gas flow of 10 L/min with 30 s plasma exposure.

### 3.3. Meteorological Information

The meteorological information on monthly rainfall from 1970 to 2019 was obtained from the Andahuaylas and Pampas meteorological station from the National Meteorological and Hydrological Service of Peru-SENAMHI database. The Andahuaylas meteorological station is located in the province of Andahuaylas at coordinates 13°39′25″ S and 73°22′15″ W, at 2933 m. Likewise, the Pampas meteorological station is located in the province of Chincheros at coordinates 13°26′5.57″ S and 73°49′27.37″ W, at an altitude of 2021 m.

For the present study and due to the nature of rainfall in the Andes, the leachate sample was taken in April for the rainy season and in June for the dry season.

### 3.4. LPI Calculation

The LPI allows for an evaluation of the level of contamination of leachates from landfills [20]. To analyze the overall LPI, 16 leachate parameters were considered (BOD_5_, COD, total coliforms (TC), pH, NH_3_N, TDS, Cl^−^, Fe, Cu, Ni, Zn, Pb, Cr, Hg, As, and CN^−^) and were divided into three sub-indexes—LPI organic (LPI_or_), LPI inorganic (LPI_in_), and LPI heavy metals (LPI_hm_)—which were calculated using Equation (1).

(1) $$\mathrm{LPI}=\;\frac{\sum_{i=1}^{m}wi\;pi\;}{\sum_{i=1}^{m}wi}$$

where LPI is the Leachate Pollution Index, *wi* is the weight of the parameter *i, pi* is the sub-index score for parameter *i,* and *m* is the number of parameters *m* < 18 *y* $\sum_{i=1}^{m}w_{i}<1$ [20]. The weight for the pollutant variable “*wi*” and the score of the sub-index curves for each pollutant “*pi*” are selected as proposed by Kumar and Alappat [20,55,58]. The LPI sub-indexes include the following parameters or pollutants:

LPI_or_ is composed of BOD_5_, COD, and TC (LPI_or_ > 7.03, which indicates a high organic pollutant load) [55,59].

LPI_in_ is composed of pH, NH_3_N, TDS, and Cl^−^ (LPI_in_ > 6.57, which indicates a high inorganic contaminant load) [55,59].

LPI_hm_ is composed of Fe, Cu, Ni, Zn, Pb, Cr, Hg, As, and CN^−^ (LPI_hm_ > 7.89, which indicates a high metal contaminant load) [55,59].

For the overall LPI, the combination of the three sub-indexes is used, using Equation (2) [20]. Values above 7.37 are considered to be indicative of potentially contaminating leachates [55,59].


(2) $${\mathrm{LPI}}_{\mathrm{ov}}=0.232\;{\mathrm{LPI}}_{\mathrm{or}}+0.257\;{\mathrm{LPI}}_{\mathrm{in}}+0.511\;{\mathrm{LPI}}_{\mathrm{hm}}$$

#### 3.5. Calculation of Discharge Volume

The daily volume of leachate generated in landfills significantly influences the LPI value [3]. The discharge volume can be estimated using Equation (3), proposed by the Australian Department of Environment, Water, Heritage and the Arts [22].

(3) $$V=\frac{0.15\times R\times A}{365}$$

where *V* is the daily leachate discharge volume (m^3^/day), *R* is the annual precipitation (m), and *A* is the surface of the landfill (m^2^).

#### 3.6. Statistical Techniques

Data were analyzed using the arithmetic mean and standard deviation. The mean difference between seasons (T1 and T2) and sampling points (L1, L2, P1, and P2) was evaluated. For this, an analysis of variance (ANOVA) was applied, and the Tukey test was applied at 5% significance. The data were processed using the Minitab V20.3 software and Excel spreadsheets version 2018.

A multivariate principal component analysis (PCA) and Pearson’s correlation (at a 5% significance level) were applied. Data processing was performed using Excel and OriginPro 2025 software.

## 4. Conclusions

The present study revealed high concentrations of organic and inorganic compounds in the ASL and AHSL. The most significant parameters in the characterization of the leachate were BOD_5_, COD, and bacteria, which showed high concentrations during the rainy season because the precipitation increased the drag of biodegradable organic matter and the microbial load in the leachates. On the other hand, parameters such as pH, NH_3_N, TDS, Cl^−^, Cr, Pb, and Hg showed high concentrations in the dry season.

Furthermore, the BOD/COD ratio in the leachates from the ASL and AHSL ranged between 0.47 and 0.74, which is characteristic of young landfills with a high biodegradable organic load. These results indicate that a significant fraction of the organic matter present in the leachates is susceptible to biological degradation. The LPI value is 30.88 for the AHSL, while it is 29.75 for the ASL, indicating that the leachate is highly contaminated and that adequate treatment should be guaranteed before discharging the leachate. Therefore, this study emphasizes that the LPI indicators are suitable to assess the degree of leachate contamination using a numerical value, as well as specifying the most critical parameters that contribute to the LPI_ov_, providing valuable information to prioritize an adequate treatment for the leachate. The sanitary landfills in the high Andean zones have leachates with a high organic matter load, which suggests that solid waste is not adequately utilized.

The landfills evaluated in this study are located in high-altitude areas near the sources of major watershed systems. This geographic setting increases the environmental risks associated with leachate contamination, as these headwater regions are ecologically fragile and vital for supplying clean water to downstream populations. The structural conditions of the leachate ponds in these locations do not ensure proper containment, allowing leachate to potentially infiltrate the soil. Likewise, during the rainy season, there is an increased risk of pond overflow or collapse, which can lead to the transport of contaminants through surface runoff or infiltration into nearby water bodies. Poor leachate management in such sensitive zones can have serious long-term effects on water quality, aquatic life, and public health. Therefore, there is an urgent need to improve landfill design and operation, strengthen environmental monitoring, and promote policies that specifically protect high-altitude hydrological systems.

## Figures and Tables

**Table 1:** Estimated volume of leachate in Andahuaylas and Ancco-Huayllo.

	Unit	ASL	AHSL
Annual Rainfall	mm/año	686.73	626.04
Waste disposal area	m^2^	24,900	6900
Discharge volume	m^3^/year	2565.95	711.05
	m^3^/day	7.03	1.95

**Table 2:** Characterization of leachate from the Andahuaylas Sanitary Landfill (ASL).

Parameters	Rainy Season (T1)						Dry Season (T2)					
	L1			L2			L1			L2		
	$\bar{x}$	±S	*	$\bar{x}$	±S	*	$\bar{x}$	±S	*	$\bar{x}$	±S	*
pH	7.268	0.010	d	7.463	0.010	c	7.641	0.010	b	7.950	0.010	a
TDS	7784.000	42.000	b	8568.000	105.000	a	8361.000	193.000	a	8621.000	23.000	a
TC	1780.000	120.000	a	1200.000	88.900	b	1230.000	147.300	b	700.000	100.000	c
COD	9446.667	35.100	a	7633.333	25.200	b	6806.667	85.100	c	5016.800	15.300	d
BOD_5_	5960.000	20.000	a	4110.000	18.000	b	3220.000	12.000	c	2470.000	5.000	d
BOD_5_/COD	0.631			0.538			0.473			0.492		
NH_3_N	647.667	5.800	b	213.333	5.800	d	1243.303	11.500	a	560.000	0.001	c
Cl^-^	2320.000	15.000	d	2400.000	26.000	c	2680.000	10.000	b	3220.000	25.000	a
Cr	0.065	0.001	c	0.061	0.001	d	0.066	0.001	b	0.068	0.001	a
Pb	0.370	0.001	c	0.317	0.001	d	0.467	0.030	b	0.510	0.020	a
Hg	0.436	0.001	c	0.414	0.001	d	0.442	0.001	b	0.451	0.000	a
As	1.748	0.040	a	1.765	0.030	a	1.466	0.030	b	1.543	0.020	b
CN^-^	0.063	0.001	a	0.002	0.001	c	0.026	0.001	b	0.003	0.001	c
Zn	0.034	0.001	c	0.007	0.001	d	0.290	0.001	a	0.275	0.001	b
Ni	0.006	0.001	a	0.004	0.001	b	0.004	0.001	b	0.004	0.001	c
Cu	0.135	0.001	a	0.140	0.001	a	0.085	0.010	b	0.081	0.010	b
Fe	1.938	0.100	b	1.970	0.100	b	4.607	0.040	a	1.837	0.010	b
Cd	0.279	0.030	b	0.184	0.010	b	1.938	0.170	a	1.970	0.170	a

Here, $\bar{x}$ is the mean, S is the standard deviation, T1 and T2 are the sampling seasons, and L1 and L2 are the leaching ponds. * indicates the significant difference in the rows evaluated by the Tukey test at 5%. N = 3.

**Table 3:** Characterization of leachates from the Ancco-Huayllo Sanitary Landfill (AHSL).

Parameters	Rainy Season (T1)						Dry Season (T2)					
	P1			P2			P1			P2		
	$\bar{x}$	±S	*	$\bar{x}$	±S	*	$\bar{x}$	±S	*	$\bar{x}$	±S	*
pH	7.848	0.010	b	7.850	0.000	b	8.346	0.240	a	8.444	0.190	a
TDS	6386.700	234.600	a	6242.700	161.900	a	7532.000	202.700	a	7614.600	294.100	a
TC	41,600.00	-		43,300.00	-		700.000	-		680.000	-	
COD	9933.3	21.000	b	10,653.3	89.600	a	9125.000	17.300	d	9315.000	8.700	c
BOD_5_	7680.000	10.000	b	7840.000	20.000	a	4830.000	10.000	c	4720.000	15.00	d
BOD_5_/COD	0.773			0.735			0.529			0.507		
NH_3_N	983.333	5.800	d	1033.333	5.800	c	1810.000	0.005	a	1470.000	0.005	b
Cl^-^	660.000	0.005	d	880.000	0.005	c	1220.000	0.005	a	960.000	0.005	b
Cr	0.118	0.001	c	0.116	0.001	d	0.172	0.001	b	0.182	0.001	a
Pb	0.205	0.010	b	0.198	0.010	b	0.370	0.020	a	0.357	0.020	a
Hg	0.339	0.001	c	0.335	0.001	c	0.396	0.001	b	0.408	0.001	a
As	1.351	0.010	c	1.331	0.020	c	1.483	0.020	a	1.433	0.010	b
CN^-^	0.001	0.001	b	0.002	0.001	a	0.001	0.001	b	0.003	0.001	b
Zn	0.025	0.001	d	0.045	0.001	c	0.379	0.001	a	0.366	0.001	b
Ni	0.005	0.001	b	0.006	0.001	a	0.002	0.001	c	0.002	0.001	d
Cu	0.081	0.001	a	0.080	0.001	a	0.089	0.010	a	0.082	0.001	a
Fe	1.106	0.100	c	1.098	0.100	c	5.419	0.020	a	4.003	0.020	b
Cd	0.098	0.010	b	0.095	0.020	b	0.199	0.001	a	0.193	0.001	a

Here, $\bar{x}$ is the mean, S is the standard deviation, T1 and T2 are the sampling seasons, P1 and P2 are the leaching ponds. * Indicate the significant difference in the rows evaluated by the Tukey test at 5%. N = 3.

**Table 4:** Sub-indexes and LPI_ov_ of the Andahuaylas Sanitary Landfill (ASL).

	Rainy Season (T1)							Dry Season (T2)					
			L1			L2			L1			L2	
	*wi*	Conc. (mg/L)	*pi*	(*pi*)(*wi*)	Conc. (mg/L)	*pi*	(*pi*)(*w*i)	Conc. (mg/L)	*pi*	(*pi*)(*wi*)	Conc. (mg/L)	*pi*	(*pi*)(*wi*)
COD	0.267	9446.667	78.00	20.83	7633.333	73.00	19.49	6806.667	70.00	18.69	5016.800	65.00	17.36
BOD_5_	0.263	5960.000	53.00	13.94	4110.000	55.00	14.47	3220.000	54.00	14.20	2470.000	43.00	11.31
TC	0.224	1780.000	75.00	16.80	1200.000	73.00	16.35	1230.000	71.00	15.90	710.000	66.10	14.81
Sum	0.754			51.57			50.31			48.80			43.47
LPI_or_				68.39			66.72			64.72			57.65
pH	0.214	7.268	5.00	1.07	7.463	4.00	0.86	7.641	4.00	0.86	7.950	3.50	0.75
NH_3_N	0.198	647.667	75.00	14.85	213.333	20.00	3.96	1243.303	100.00	19.80	560.000	60.00	11.88
TDS	0.195	7784.00	15.50	3.02	8568.000	17.00	3.32	8361.000	17.00	3.32	8621.000	18.30	3.57
Cl^−^	0.187	2320.00	16.10	3.01	2400.000	18.00	3.37	2680.000	20.00	3.74	3220.000	26.00	4.86
Sum	0.794			21.95			11.50			27.71			21.06
LPI_in_				27.65			14.48			34.90			26.52
Cr	0.125	0.065	5.00	0.63	0.061	5.00	0.63	0.066	5.00	0.63	0.068	5.00	0.63
Pb	0.123	0.370	6.00	0.74	0.317	6.00	0.74	0.467	6.00	0.74	0.510	7.00	0.86
Hg	0.121	0.436	45.10	5.46	0.414	50.00	6.05	0.442	51.00	6.17	0.451	50.00	6.05
As	0.119	1.748	12.00	1.43	1.765	12.00	1.43	1.466	10.00	1.19	1.543	11.00	1.31
CN^−^	0.114	0.063	5.00	0.57	0.002	5.00	0.57	0.026	5.00	0.57	0.003	5.00	0.57
Zn	0.11	0.034	5.00	0.55	0.007	5.00	0.55	0.290	5.00	0.55	0.275	5.00	0.55
Ni	0.102	0.006	5.00	0.51	0.004	5.00	0.51	0.004	5.00	0.51	0.004	5.00	0.51
Cu	0.098	0.135	5.00	0.49	0.140	5.50	0.54	0.085	5.00	0.49	0.081	5.00	0.49
Fe	0.088	1.938	5.00	0.44	1.970	5.00	0.44	4.607	5.00	0.44	1.837	5.00	0.44
Sum	1.000			10.81			1145.00			10.81			11.41
LPI_hm_				10.81			11.45			11.28			11.41
LPI_ov_				28.50			24.81			29.75			26.02

Here, L1 and L2 are sampling points, “*wi*” weight of the contaminant variable, “*pi*” score of the subscript curves for each contaminant [20,55,58].

**Table 5:** Sub-indexes and LPI_ov_ of the Ancco-Huayllo Sanitary Landfill (AHSL).

	Rainy Season (T1)							Dry Season (T2)					
			P1			P2			P1			P2	
	*wi*	Conc. (mg/L)	*pi*	(*pi*)(*wi*)	Conc. (mg/L)	*pi*	(*pi*)(*w*i)	Conc. (mg/L)	*pi*	(*pi*)(*wi*)	Conc. (mg/L)	*pi*	(*pi*)(*wi*)
COD	0.267	9933.30	74.50	19.89	10,653.30	75.00	20.03	9125.00	74.00	19.76	9315.00	74.00	19.76
BOD_5_	0.263	7680.000	63.00	16.57	7840.000	63.50	16.70	4830.000	56.00	14.73	4720.000	55.00	14.47
TC	0.224	41,600.00	96.50	21.62	43,300.00	97.00	21.73	700.000	66.50	14.90	680.000	66.00	14.78
Sum	0.754			58.08			58.45			49.38			49.01
LPI_or_				77.03			77.53			65.49			65.00
pH	0.214	7.848	4.00	0.86	7.850	4.00	0.86	8.346	5.00	1.07	8.444	5.00	1.07
NH_3_N	0.198	983.333	99.00	19.60	1033.333	100.00	19.80	1810.000	100.00	19.80	1470.000	100.00	19.80
TDS	0.195	6386.700	14.10	2.75	6242.70	14.00	2.73	7532.000	16.00	3.12	7614.600	16.10	3.14
Cl^−^	0.187	660.000	7.50	1.40	880.000	8.00	1.50	1220.000	9.00	1.68	960.000	8.30	1.55
Sum	0.794			24.61			24.88			25.67			25.56
LPI_in_				31.00			31.34			32.33			32.19
Cr	0.125	0.118	5.00	0.63	0.116	5.00	0.63	0.172	5.00	0.63	0.182	5.00	0.63
Pb	0.123	0.205	5.00	0.62	0.198	5.00	0.62	0.370	6.30	0.77	0.357	6.30	0.78
Hg	0.121	0.339	38.00	4.60	0.335	37.00	4.48	0.396	44.00	5.32	0.408	45.00	5.45
As	0.119	1.351	10.00	1.19	1.331	10.00	1.19	1.483	11.00	1.31	1.433	11.00	1.31
CN^−^	0.114	0.001	5.00	0.57	0.002	5.00	0.57	0.001	5.00	0.57	0.003	5.00	0.57
Zn	0.11	0.025	5.00	0.55	0.045	5.00	0.55	0.379	5.00	0.55	0.366	5.00	0.55
Ni	0.102	0.005	5.00	0.51	0.006	5.00	0.51	0.002	5.00	0.51	0.002	5.00	0.51
Cu	0.098	0.081	5.00	0.49	0.080	5.00	0.49	0.089	5.00	0.49	0.082	5.00	0.49
Fe	0.088	1.106	5.00	0.44	1.098	5.00	0.44	5.419	5.00	0.44	4.003	5.00	0.44
Sum	1			9.59			9.47			10.59			10.71
LPI_hm_				9.59			9.47			10.59			10.71
LPI_ov_				30.74			30.88			28.92			28.83

Here, P1 and P2 are sampling points, “*wi*” is the weight of the contaminant variable, and “*pi*” is the score of the subscript curves for each contaminant [20,55,58].

**Table 6:** Comparison of the Leachate Pollution Index.

N°	Location	Type	Country	Status	LPI Value	Reference
1	Vissershok Landfill	Sanitary Landfill	África	Active	46.55	[3]
2	Varanasi Landfill	Dump	India	Active	37.91	[3]
3	Turbhe Landfill	Sanitary Landfill	India	-	36.83	[65]
4	Sungai Sedu Landfill	Dump	Malaysia	Active	35.13	[3]
5	Dhapa Landfill, Kolkata	Dump	India	Active	34.02	[59]
6	Chandigarh Landfill	Dump	India	-	33.18	[39]
7	Okhla Landfill, New Delhi	Sanitary Landfill	India	-	32.50	[60]
8	Brahmapuram Landfill, Kochi	Composting Plant	India	Active	31.99	[53]
9	Ancco-Huayllo Sanitary Landfill	Sanitary Landfill	Perú	Active	30.88	(Present study)
10	Andahuaylas Sanitary Landfill	Sanitary Landfill	Perú	Active	29.75	(Present study)
11	SAS Nagar Landfill	Dump	India	-	26.17	[39]
12	Mavallipura Landfill	Municipal Sanitary Landfill	India	-	25.10	[66]
13	Pune Dumping Site	Dump	India	Active	24.67	[67]
14	Air Hitam Sanitary Landfill	Sanitary Landfill	Malaysia	Active	24.63	[3]
15	Nam Son Landfill, Hanoi	Sanitary Landfill	Vietnam	Active	24.60	[10]
16	Ikhueniro Landfill, Benin City, Edo State	Dump	Nigeria	Active	22.31	[64]

**Table 7:** State of sanitary landfills.

Situation	ASL	AHSL
State	New—Active	New—Active
Operation period	4 years 10 months	5 years
Landfill area	96,173.87 m^2^	26,167.09 m^2^
Waste disposal area	≈24,900 m^2^	≈6900 m^2^
Waste type	Municipal waste	Municipal waste
Topography	Steep slope	Inclined slope
Soil type	Clay silt	Clay loam
Quantity of solid waste (t/day) ^a^	24.31 t/day	6.46 t/day
Quantity of solid waste (t/year) ^a^	16,578.90 t/years	2042.07 t/years
Bottom coating	Yes	No
Leachate treatment	Yes	No
Leachate volume discharged	NA ^b^	NA ^b^

^a^ SIGERSOL [68]; NA ^b^—information not available.

**Table 8:** Sources and quantity of solid waste disposal in 2022.

ASL		AHSL	
Districts	Metric Ton (t)	Districts	Metric Ton (t)
Andahuaylas	11,454.94	Ancco-Huyallo	2042.07
San Jerónimo	2560.66		
Talavera	2342.18		
Pacucha	221.12		
Total	16,578.90	Total	2042.07

Source: SIGERSOL [68].

**Table 9:** Parameter analysis methods.

Parameter	Method	Unit	Reference
Biochemical Oxygen Demand (BOD_5_)	Respirometric, Manometric oxytope method	mg O_2_/L	Standard methods 5210D [70]
Chemical Oxygen Demand (COD)	Closed Reflux, Colorimetric Method	mg O_2_/L	Standard methods 5220B [70]
Total Coliforms (TC)	*E. coli*/Coliform Count	CFU/ 100 mL	3M Petrifilm Plates [71]
pH	Selective electrode	-	Standard methods 4500 – H + B [70]
Ammonia Nitrogen (NH_3_-N)	Nessler Method	mg/L	ASTM Standards, D 1426 [72]
Total Dissolved Solids (TDS)	Gravimetric Method	mg/L	Standard methods 2540 B [70]
Total Suspended Solids (TSS)	Gravimetric Method	mg/L	Standard methods 2540 B [70]
Chloride (Cl^−^)	Mercury (II) thiocyanate method	mg/L	HANNA Instruments [72]
Iron (Fe)	Optical Emission Spectrometry	mg/L	
Copper (Cu)			ICP-OES 9820
Cadmium (Cd)			
Zinc (Zn)			
Lead (Pb)			
Total Chromium (Cr)			
Mercury (Hg)			
Arsenic (As)			
Nickel (Ni)			
Cyanide (CN^−^)	Pyridine–Pyrazalone method	mg/L	HANNA Instruments [72]

## Data Availability

The data presented in this study are available in the article.
